# Measuring and Enhancing Initial Parent Engagement in Parenting Education: Experiment and Psychometric Analysis

**DOI:** 10.2196/37449

**Published:** 2022-09-30

**Authors:** Isaac A Mirzadegan, Amelia C Blanton, Alexandria Meyer

**Affiliations:** 1 Department of Psychology Florida State University Tallahassee, FL United States

**Keywords:** parental engagement, parenting intervention, parenting education, intent to enroll, measure development

## Abstract

**Background:**

Prevention efforts focused on parenting can prevent and reduce the rates of child internalizing and externalizing problems, and positive changes in parenting skills have been shown to mediate improvements in child behavioral problems. However, parent skills training programs remain underused, with estimates that under half of eligible parents complete treatment and even lower rates engage in preventive interventions. Moreover, there is no validated measure to assess initial engagement in parent education or skills training, which is an understudied stage of parent engagement.

**Objective:**

We aimed to test a novel engagement strategy, exploring whether including information pertaining to the neuroscience of child development and parent skills training enhanced parental intent to enroll. In addition, a novel self-report measure, the 18-item Parenting Resources Acceptability Measure (PRAM), was developed and validated.

**Methods:**

In a group of 166 parents of children aged 5 to 12 years, using an engagement strategy based on the *Seductive Allure of Neuroscience Explanations*, we conducted a web-based experiment to assess whether the inclusion of neuroscience information related to higher levels of engagement via self-report and behavioral measures. The PRAM was subjected to an exploratory factor analysis and examined against relevant validity measures and acceptability measurement criteria.

**Results:**

Three PRAM factors emerged (“Acceptability of Parenting Resources,” “Interest in Learning Parenting Strategies,” and “Acceptability of Parenting Websites”), which explained 68.4% of the total variance. Internal consistency among the factors and the total score ranged from good to excellent. The PRAM was correlated with other relevant measures (Parental Locus of Control, Parenting Sense of Competence, Strengths and Difficulties Questionnaire, Parent Engagement in Evidence-Based Services, and behavioral outcomes) and demonstrated good criterion validity and responsiveness. Regarding the engagement manipulation, parents who did not receive the neuroscience explanation self-reported lower interest in learning new parenting skills after watching an informational video compared with parents who did receive a neuroscience explanation. However, there were no significant differences between conditions in behavioral measures of intent to enroll, including the number of mouse clicks, amount of time spent on a page of parenting resources, and requests to receive parenting resources. The effects did not persist at the 1-month follow-up, suggesting that the effects on engagement may be time-limited.

**Conclusions:**

The findings provide preliminary evidence for the utility of theory-driven strategies to enhance initial parental engagement in parent skills training, specifically parental interest in learning new parenting skills. In addition, the study findings demonstrate the good initial psychometric properties of the PRAM, a tool to assess parental intent to enroll, which is an early stage of engagement.

## Introduction

### Parent Engagement in Parenting Education and Skill Training

Promoting child mental health has been identified as a key public health issue [1-3]. Most forms of child mental health treatment involve parents, with many efficacious interventions even focusing exclusively on parents, most commonly in the form of parent skills training [4,5]. Parent-based skill training, or parenting education, can take the form of parenting groups, individual treatment, self-help (such as parenting books), and web-based programs. A wealth of evidence suggests that parent skills training programs are effective in reducing child internalizing and externalizing symptoms [6-9]. In addition, parenting education with a *prevention* focus has been shown to reduce the risk of a wide range of youth emotional and behavioral problems over follow-up periods of up to 20 years [10]. Thus, parent skills training programs are clearly an effective way to treat and prevent child mental health problems.

Despite the demonstrated efficacy of parenting education programs, low parental engagement in these programs has been recognized as a significant barrier to improving child mental health [11]. A systematic review of parental engagement found that 25% of parents who met the criteria for behavioral parent training interventions did not enroll in treatment, and an additional 26% dropped out before the end of treatment, leading to 51% of identified eligible parents not completing treatment [12]. Thus, most families who may stand to benefit from parent training services do not receive a full dose of treatment. Engagement rates in preventive parenting interventions have been found to be similarly low or even lower than those in standard interventions [13,14].

Unfortunately, limited engagement can compromise the ability of parenting education programs to provide desired outcomes for children and families and can temper conclusions drawn from parent skills training research [15]. Moreover, programs with underenrollment are less cost-effective, limiting the effectiveness and disseminability of parenting intervention programs [16]. A host of parent-level, community-level, and programmatic factors have been associated with lower levels of engagement in prevention programs, including socioeconomic disadvantage, lack of social support, single parent status, and minority status [13]; younger parental age and neighborhood disadvantage [11]; parental attributions (eg, external locus of control regarding child behaviors) [17,18]; and limited parental knowledge of efficacious child treatments [19]. A recent study also examined factors that positively influence intent to engage in a parenting intervention in a very large web-based sample (N=6733). Parent behaviors (ie, more coercive parenting, lower consistency, greater use of positive encouragement, and more help-seeking behaviors) and parent cognitions (ie, lower sense of parental self-efficacy, greater psychological distress, and lower perceived quality of parent-child relationship) emerged as significant positive predictors of intent to engage in a parenting program [18].

### Methods to Enhance Engagement

Piotrowska et al [20] outlined a model of parent engagement with 4 stages: connection, attendance, participation, and enactment. Connection, the earliest stage of engagement, occurs when parents decide to enroll. Importantly, intent to enroll significantly predicts subsequent enrollment and is a moderate-strength predictor of first-session attendance [21,22]. However, despite the underenrollment of parents in prevention and intervention programs to promote child mental health, few studies have experimentally investigated methods to increase program engagement, with only a handful of methods demonstrating efficacy in the early stages of parent engagement. These have included a promotion-focused advertisement [23], a comprehensive “engagement package” (ie, a testimonial flyer, teacher endorsement, and extra calls from group leaders) [24,25], and monetary incentives [26,27].

Overall, few experimental studies have attempted to increase initial engagement. Furthermore, studies have used inconsistent operationalizations of the different aspects of engagement, experimental studies tend to have low methodological quality, and the diversity of methods to enhance engagement makes generalizations difficult [28,29]. Moreover, most initial engagement knowledge comes from intervention research that examined recruitment and enrollment factors post hoc. In these designs, only information from parents who were interested in enrolling is accessible, which precludes identifying the factors associated with parents who choose *not* to enroll. Taken together, these reviews suggest that there is a need to test novel methods to promote initial parental engagement in preventive interventions.

A separate line of work suggests that including neuroscience explanations can increase subjective credibility and favorability ratings of written information; this phenomenon is known as the “Seductive Allure of Neuroscience Explanations” (SANE) [30,31]. For example, explanations are viewed as more compelling if they include statements such as “brain scans show that...” or “frontal lobe brain circuitry is known to be involved in...” [30,31]. Some researchers have found that this effect is driven by mere conceptual inclusion of neuroscience information [32], whereas others have found this effect only when superfluous neuroscience text *and* images are included [33]. A review of the neuroimage bias literature concluded that the effects of including these superfluous images may vary according to contextual characteristics [34]. Furthermore, it has been proposed that using optimistic neuroscience explanations that characterize the brain as neuroplastic may enhance the credibility of the information and promote positive social-emotional responses within an intervention [35]. Although it has been suggested that neuroscience explanations may increase engagement with treatment, to our knowledge, this has not been demonstrated experimentally.

In this study, considering the potential utility of preventive parent training, we examined whether a neuroscience-enhanced video presentation about child development and parent skills training enhanced parental intent to enroll more than a program description without neuroscience (standard video). Focusing on the initial stage of engagement, *connection* [20], we directly compared 2 different methods of advertising a preventive parent skills training program, maximizing the data collected from both interested *and* uninterested parents. Our model integrates 2 separate lines of work: one on the impact of neuroscience explanations on credibility and the other on parent intervention engagement. We used a framework consistent with the SANE aiming to expand the menu of engagement strategies. Moreover, this study examined the initial stages of engagement in depth, collecting information on self-reported interest in addition to capturing behavioral proxies of intent to enroll.

### Measuring Initial Engagement

Beyond the limitations of using self-selected samples of caregivers in initial engagement research, there is a lack of measures of engagement at this earliest stage. In the context of parents already enrolled in a behavioral parent training program, the Parent Motivation Inventory [36] assesses parent desire for child change along with readiness and perceived ability to change parenting behaviors. Other measures have been developed to assess conceptually related constructs, such as the Parental Attitudes Toward Psychological Services Inventory [37] and the Parent Engagement in Evidence-Based Services (PEEBS) questionnaire [38,39], both of which assess parental attitudes toward mental health services. Importantly, these measures assess parental openness to engaging their child in treatment rather than the acceptability of *parent-focused* training.

In light of this measurement gap and the goal of this study to examine the impact of neuroscience information on parent willingness to engage with parenting education materials, we developed and validated a measure to assess parental intent to enroll, part of the *connection* stage of engagement [20]. The Parenting Resources Acceptability Measure (PRAM; Multimedia Appendix 1) was created based on a measure of acceptability of positive parenting strategies [40] and on previous work examining consumer preferences for parenting support delivery methods [41]. This measure was developed and evaluated according to a set of established criteria for acceptability measures [42]. Following the development of the 18 items, the measure validation proceeded in 3 phases. First, the measure was subject to an exploratory factor analysis (EFA), and the factors and total scores were correlated with established measures of other relevant constructs. Validity measures were selected based on published measures assessing conceptually related constructs [38,39] along with constructs that have been shown to relate to parent engagement, including child problems [43,44], social support [13], parental locus of control [17], and parental self-efficacy [18]. Next, this measure was used in the experimental study outlined previously. Finally, a subsample of parents was assessed 1 month following the baseline battery administration, and the PRAM was correlated with self-reported engagement in parenting education resources in the previous month. These findings represent the initial validation of this novel measure of the acceptability of parenting education resources.

We hypothesized that the PRAM would demonstrate strong psychometric properties and relate to theoretically relevant measures. We further hypothesized that a neuroscience-enhanced video presentation about child development and parent skills training would enhance parental intent to enroll, as measured by the PRAM, compared with a presentation without neuroscience information. We also hypothesized that this group would display behavior consistent with higher intent to enroll, operationalized as more mouse clicks and time spent on a page of parenting resources, along with a greater likelihood of requesting parenting resources following the presentation.

## Methods

### Participants and Procedure

#### Recruitment and Study Completion

Participants were drawn from a registry of families previously recruited for research by the Center for Developmental Science of the Psychology Department at Florida State University. Potential participants were not selected based on pre-existing traits or risk factors; thus, this study is consistent with a *universal prevention* approach [45]. Caregivers were called and asked to participate in a study examining attitudes, interests, and the impact of COVID-19 on Tallahassee families. Participants were compensated with a US $10 Amazon gift card for participating in the baseline survey and entered to win a US $150 gift card for completing the 1-month follow-up survey. The eligibility criteria were as follows: (1) being a parent or legal guardian, and primary caregiver, of at least one child aged 5 to 12 years; (2) having access to a computer; (3) currently living in Tallahassee; and (4) being able to respond to questions in English. Though the term “parent” is used throughout this paper, this term includes nonparent legal guardians.

Participants were then randomly assigned to one of two conditions, with an equal chance of being in either condition, and emailed a link to an approximately 1-hour–long survey (Qualtrics XM Platform; Qualtrics International Inc), which they were instructed to complete in a single sitting and within 1 week. However, survey responses collected beyond the 1-week time limit were included; the purpose of stating a time limit was to increase participation rates. Reminder emails or calls were sent or given 5 days after the initial enrollment and every 1 to 2 weeks thereafter. The purpose of instructing parents to complete the survey in a single sitting was to reduce the likelihood of a disruption occurring during the video manipulation. At the end of the survey, participants were asked about any interruptions. Although 31.2% (53/170) of parents indicated that they experienced a disruption during the survey, participants were only excluded if the disruption occurred during the video manipulation (4/170, 2.4%). Finally, a list of parenting resources was sent in a follow-up email upon request (ie, “Would you like to be emailed a list of parenting resources after this survey?”).

Follow-up surveys were emailed to caregivers approximately 1 month after completion of the baseline survey. In this survey, participants were readministered a subset of measures included in the baseline survey, and they were asked whether they had engaged with any of the services or resources provided after the baseline survey.

#### Final Sample

A total of 590 family registry phone numbers were called. Of these 590 phone numbers, 108 (18.3%) were deemed ineligible (eg, had moved away or had no children in the age range) or were unable to be contacted (eg, phone number disconnected), 164 (27.8%) were left voicemails that were never returned, 32 (5.4%) were reached but unwilling or unable to speak in the moment and not reached again later, and 58 (9.8%) expressed that they were not interested. A total of 228 parents agreed by phone to participate in the study and were emailed a link to the survey. Of these parents, 76.3% (174/228) completed the entire baseline survey, 6.6% (15/228) partially completed the survey, 0.9% (2/228) unenrolled from the study (1/2, 50% lost access to a computer and 1/2, 50% unenrolled for medical reasons), and 16.2% (37/228) never started the survey. Of the 174 parents who completed the survey, 4 (2.3%) reported experiencing a disruption during the video presentation and were excluded. Finally, all parents who reported being nonprimary caregivers (4/174, 2.3%) were excluded. Thus, the final sample consisted of 166 parents, with equal numbers per condition (83/166, 50%).

Of the 166 parents who completed the baseline survey, 128 (77.1%) completed the follow-up survey regarding past-month use of parenting resources, completing it an average of 38 (SD 21) days after the baseline survey. Follow-up completers did not differ from the noncompleters in any demographic characteristic or PRAM scores at baseline (*P*>.05 in all cases).

Owing to an early administrative error, 34% (28/83) of parents who had been randomly assigned to the standard video were erroneously sent the neuroscience-enhanced video survey link, leading to an imbalance of numbers per condition. Recruitment goals were extended to balance condition assignment and, thus, the conditions were pseudorandomized. However, the timing of enrollment did not relate to any study variables, suggesting that the groups likely did not differ systematically as a result of this error. Moreover, follow-up survey completion rates did not differ between participants who were truly randomized and those who were pseudorandomized to either the standard video or neuroscience-enhanced video.

### Manipulation Materials

The baseline Qualtrics survey included a number of questionnaires presented in a randomized order, followed by an experimental manipulation; a page of parenting resources with which parents could interact; and, finally, the postmanipulation repeated measure.

#### Video Presentations

The experimental manipulation consisted of a simple, narrated slideshow video presentation with textual captions on-screen. The video presentation came in 2 formats: standard video (60 seconds) and neuroscience-enhanced video (97 seconds). These conditions were identical besides the additional content in the neuroscience-enhanced video focused on the effect of parenting on child brain development. To avoid the confound of adding extraneous content to the standard video condition, video length was confounded with video content (video links are available in Multimedia Appendix 2 [46-55]).

The videos were designed to be similar in language to what is advertised in currently available positive parenting resources, as determined by the first author’s informal survey of popular web-based parenting resource web pages and course offerings. Video content was developed following stakeholder input from a parent of 2 young children who also directed a nonprofit organization providing parenting resources and education. Consultation focused on identifying appropriate and compelling terminology (eg, “effective parenting” and “supporting child development”). Additional input was solicited from a panel of psychologists, many of whom specialized in child development and were themselves parents of young children. On the basis of previous research that favors a health *promotion* focus over a problematic behavior *prevention* focus, the videos emphasized health promotion [23]. The lead author’s voice was used for the slideshow narration.

The standard video design included general information about parent skills training programs and resources and how they can be helpful for promoting healthy child development and effective and positive parenting, addressing behavioral or emotional problems, decreasing parenting stress, and increasing feelings of parenting efficacy. It included descriptions of raising children along with images of happy families.

The neuroscience-enhanced video design included identical content to the standard video, with 2 additional slides of general information about how children’s development can be mapped out in the brain and how parenting behaviors, and parent skills training in particular, can affect neurodevelopmental trajectories. It also featured 2 images depicting children’s brains.

Immediately after viewing the video (standard video or neuroscience-enhanced video), participants were asked the following: “Would you like to learn more about resources for parents on positive parenting practices?” Regardless of the response, all parents were then taken to the resource page within the survey.

#### Resources and Program Links

A page with 8 parenting resource websites was displayed. Resources included web-based parent skills training courses (a free, 4-week, web-based parenting course [*Everyday Parenting* by Kazdin] and a US $80 web-based parenting skills class), in-person or local parenting resources (a parenting resource page from a local Early Learning Coalition and a community resource directory page from a pediatric health organization), self-help written parenting resources [46,47], and web pages with evidence-based parenting information (*Positive Parenting Tips* from the Centers for Disease Control and Prevention and *Resources for Families* from the Child Mind Institute). Multimedia Appendix 2 contains further details on these resource pages. The purpose of providing these web pages was to measure parents’ behavioral engagement, including mouse clicks and time spent on the resource page. Each resource was an embedded web page (ie, an inline frame or “iframe”) within the Qualtrics survey. Only the front page or cover of each resource was shown so that participants remained in the Qualtrics survey while navigating the resource page. However, participants were able to click and scroll within the embedded web pages. After spending as much time as desired on the resource page, participants completed the postmanipulation survey questions.

### Measures

#### Engagement Measure: PRAM

Parents completed a measure designed to assess their openness to, interest in, and likelihood of engaging in parent training (Multimedia Appendix 1). This measure was created because no measure for this construct currently exists. It was modeled after the Parenting Strategies Questionnaire, which examines parents’ rated acceptability and usefulness of and behavioral intention to engage in parenting strategies learned in a positive parenting training program for children diagnosed with autism spectrum disorder [40]. This questionnaire was chosen as the model for measuring development because of its high topical relevance and because it showed strong psychometric properties and achieved a high rating on a set of established criteria for measures of acceptability and appropriateness [42]. The PRAM has 6 general statements about parent attitudes toward receiving resources to increase knowledge of effective parenting skills and strategies, half of which are reverse-scored. In addition, consistent with the 3 proposed subscales of the Parenting Strategies Questionnaire, the PRAM contains statements about parents’ (1) rated acceptability (openness); (2) rated usefulness (interest); and (3) self-assessed behavioral intent (likelihood) to participate in specific types of parenting interventions, including a web-based parenting program, websites with information about positive parenting, local resources for parents, and books about parenting. These 4 domains mapped onto the types of resources provided to parents following completion of the PRAM. Respondents could also write other unlisted resources that they would find acceptable. All items were measured on a 5-point Likert scale (1=*strongly disagree*, 2=*disagree*, 3=*neither agree nor disagree*, 4=*agree*, and 5=*strongly agree*). The measure was subjected to an EFA and internal consistency analysis. A total mean score was also computed. Parents completed this measure at baseline, again after viewing the video manipulation, and again at the 1-month follow-up.

#### Demographics

Demographic information included the following: parent gender identity, age, education level, single or dual parent status, marital status, race or ethnicity, and income bracket; percentage of time providing childcare; number and ages of children; and previous participation in parenting classes or use of parenting self-help resources.

#### Convergent Validity

These measures were completed at baseline.

##### Child Behavioral and Internalizing Problems

Parents were given the Strengths and Difficulties Questionnaire (SDQ) to self-report their child’s internalizing and externalizing problems [56]. The SDQ is a 25-question, widely used measure of internalizing, externalizing, and prosocial behaviors, and it has satisfactory psychometric properties [57]. A 3-factor solution has been shown to be appropriate for community samples, consisting of a total difficulties score along with externalizing and internalizing subscales [58]. In addition, the total difficulties score of the SDQ has been validated as a dimensional measure of child mental health [59]. Parents were told to answer these questions as they pertained to their child with the most behavioral or emotional problems within the age range of 5 to 12 years. Higher scores reflect greater problems.

##### Perceived Social Support

The 23-item Social Support Appraisals Scale measures perceived social support on a 4-point Likert scale (1=*strongly agree* to 4=*strongly disagree*) with good reliability and adequate validity [60]. Higher scores reflect lower perceived social support. It indexes subjective experience of support from family and friends, with items such as “My family cares for me very much” and “My friends and I have done a lot for one another.” In a previous study, parent-perceived social support predicted a higher likelihood of enrollment in a prevention program but did not further distinguish families who attended at least one session [13]. Thus, social support may have the strongest predictive power in the very early stages of parent engagement.

##### Perceived Parenting Self-efficacy

The 17-item Parenting Sense of Competence (PSOC) scale [61] is measured on a 6-point Likert scale (1=*strongly agree* to 6=*strongly disagree*). It has a 3-factor structure (ie, Satisfaction [eg, “Being a parent makes me tense and anxious”], Efficacy [eg, “If anyone can find the answer to what is troubling my child, I am the one”], and Interest [eg, “My talents and interests are in other areas, not in being a parent”]), each with acceptable internal consistency in both mothers and fathers [62]. Higher scores reflect lower levels of satisfaction, efficacy, and interest. The PSOC is one of the most widely used assessments of perceived parenting self-efficacy [63].

##### Parental Knowledge and Attitudes Toward Evidence-Based Care

The PEEBS measure was developed based on the theory of planned behavior [64] and assesses parental attitudes toward and knowledge of how to engage in evidence-based care [38,39]. It is rated on a 5-point Likert scale (1=*strongly disagree* to 5=*strongly agree*). A total of 12 items comprising 2 subscales of a revised version of the PEEBS were administered, which were previously found to have acceptable reliability: Subjective Norms (Cronbach *α*=.76) and Knowledge (Cronbach *α*=.73) [39]. The Knowledge scale reflects parents’ knowledge of child-focused mental health treatments and how to access them (eg, “I know how to access treatments for my child”). The Subjective Norms scale reflects the degree to which parents generally value the endorsements of others (including from a therapist, school staff, pediatricians, psychiatrists, the web, other families, and parent advocates) in selecting a treatment for their child (eg, “Treatments endorsed by a therapist are important to me”). Higher scores reflect greater knowledge and valuation of subjective norms.

##### Parental Locus of Control

Parents completed a 36-item measure on a 5-point Likert scale (1=*strongly disagree* to 5=*strongly agree*) assessing locus of control with respect to parenting skills and behavior (Parental Locus of Control [PLOC]) [65]. This measure yields 4 factors with acceptable to good internal consistency (Parental Efficacy, where higher values reflect feeling *ineffective* in the parenting role; Parental Responsibility, where higher values reflect parents who *do not* feel responsible for their child’s behavior; Child Control, where higher scores reflect parents’ belief that their child’s needs dominate their life; and Parental Control, where higher values reflect parents who believe they are *unable* to control their child’s behavior). Example items include “I always feel in control when it comes to my child” and “I am responsible for my child’s behavior.”

#### Behavioral Measures: Additional Convergent Validity

Behavioral outcomes were used as measures of convergent validity for the PRAM as well as outcome measures for the experimental manipulation. All behavioral measures except for the 1-month follow-up retrospective report were administered immediately following the experimental manipulation within the baseline survey.

##### Request for Additional Information

The groups were examined for differences in whether they requested additional information on positive parenting resources immediately following the video presentation.

##### Mouse Clicks and Time Spent on Resource Pages

The total elapsed time, along with the number of mouse clicks, viewing the resource page within Qualtrics was recorded.

#### Predictive Validity: Follow-up Report of Behavioral Engagement

At the approximately 1-month follow-up survey, participants were asked whether they had engaged with any parenting resources in the previous month in the form of books, websites, courses, or local resources. The variable of interest was whether parents endorsed having engaged in *any* form of parenting education in the previous month.

### Statistical Analyses

To determine the validity and factor structure of the PRAM (Multimedia Appendix 1), the measure was subjected to an EFA and internal reliability analysis (ie, the Cronbach *α*). A 3-factor solution was expected, comprising one factor of acceptability (openness), one factor of rated usefulness (interest), and another factor measuring behavioral intent (likelihood).

Pearson product-moment correlations (*r*) were used to determine the strength of the relationships among PRAM factors, validity measures, and behavioral outcomes, including retrospective reports of engagement at the 1-month follow-up.

Regarding group differences based on manipulation, chi-square tests examined group differences in whether participants responded “yes” to the question, “Would you like to learn more about resources for parents on positive parenting practices?”

To test for differences in engagement between groups immediately following the manipulation within the baseline survey, a negative binomial regression compared the number of total mouse clicks between groups (neuroscience-enhanced video and standard video), and a one-way ANOVA compared the total time spent on the resource page between the 2 groups (neuroscience-enhanced video and standard video). In addition, repeated-measure (RM) ANOVAs assessed differences in acceptability before and after viewing the videos. One evaluated the pre-post differences in total PRAM scores, and 3 additional RM ANOVAs were conducted on the subfactors of this scale based on the results of the EFA.

Finally, univariate RM ANOVAs using all 3 time points of the PRAM were conducted on the subset of participants who completed the 1-month follow-up survey to assess the prospective differences between the standard video and neuroscience-enhanced video groups.

### Ethics Approval

The Florida State University Institutional Review Board exempted this study on February 26, 2020 (reference STUDY00001059).

## Results

### Preliminary Analysis

Chi-square tests and one-way ANOVAs revealed no differences in demographic variables per condition (standard version and neuroscience-enhanced version; *P*>.05 in all cases). Pre-existing differences between groups on the outcome and validity variables at baseline were ruled out using 2-tailed independent-sample *t* tests (*P*>.05). The descriptive statistics for the study participants and baseline measures are reported in Table 1 and Table 2, respectively.

Internal consistency analysis was conducted for all variables. To reduce the total number of statistics, increase the reliability of the results, and simplify interpretation, scales with poor internal consistency (ie, SDQ Internalizing Problems, PLOC Child Control, PLOC Parental Efficacy, and PSOC Interest) were omitted from further correlational analyses. Table 3 presents a bivariate correlation matrix of the important study variables.

**Table 1:** Participant demographics (N=166).

Variable			Values
**Parent demographics**			
	Age (years), mean (SD; range)		38.93 (6.38; 24-61)
	Number of children, mean (SD; range)		2.45 (1.13; 1-8)
	**Education level, n (%)**		
		High school diploma or equivalent	2 (1.2)
		Some college	17 (10.2)
		Associate degree or vocational degree	13 (7.8)
		Bachelor’s degree	56 (33.7)
		Master’s degree	57 (34.3)
		Doctorate or professional degree	21 (12.7)
	**Current employment, n (%)**		
		Unemployed	46 (27.7)
		Employed part time	21 (12.7)
		Employed full time	99 (59.6)
	**Annual household income (US $), n (%)**		
		10,000 to 39,999	14 (8.4)
		40,000 to 69,000	40 (24.1)
		70,000 to 99,000	40 (24.1)
		100,000 to 149,000	46 (27.7)
		≥150,000	26 (15.7)
	**Household structure, n (%)**		
		Dual parent	150 (90.4)
		Single parent	16 (9.6)
	**Gender, n (%)**		
		Cisgender female	153 (92.2)
		Cisgender male	11 (6.6)
		Transgender female	2 (1.2)
	**Race, n (%)**		
		White	133 (80.1)
		Black or African American	16 (9.6)
		Asian	10 (6)
		Multiracial^a^	5 (3)
		Middle Eastern or North African	1 (0.6)
		No response	1 (0.6)
	**Ethnicity, n (%)**		
		Hispanic or Latino	8 (4.8)
		Not Hispanic or Latino	158 (95.2)
	**Previous use of parenting resources^b^, n (%)**		
		Yes	110 (66.3)
		No	56 (33.7)
	**Marital status, n (%)**		
		Married	141 (84.9)
		Single or never married	11 (6.6)
		Divorced	10 (6)
		Separated	3 (1.8)
		Widowed	1 (0.6)
**Child demographics^c^**			
	Age of child (years), mean (SD; range)		8.32 (2.21; 5-12)
	**Sex of child, n (%)**		
		Male	88 (53)
		Female	77 (46.4)
		Missing	1 (0.6)
	**Schooling, n (%)**		
		Public school	118 (71.1)
		Private school	34 (20.5)
		Homeschooled	14 (8.4)

^a^Participants were able to select multiple races; thus, “multiracial” reflects participants who selected more than one race.

^b^This question explicitly excluded parenting resources related only to *childbirth*.

^c^Parents were asked to report information for their child aged 5 to 12 years with the most significant behavioral or emotional problems.

**Table 2:** Descriptives of validity measures (N=166).

Validity variable				Cronbach *α*^a^		Value, mean (SD; range)
**SDQ^b^**						
	**Total difficulties**		.81		11.76 (5.74; 2-28)	
		Externalizing symptoms	.81		7.54 (3.92; 0-20)	
		Internalizing symptoms	.67		4.22 (2.96; 0-12)	
	Prosocial scale		.77		7.43 (2.12; 1-10)	
	Parent-reported impact		—^c^		1.32 (2.10; 0-10)	
**PLOC^d^**						
	Parent control		.82		2.63 (0.73; 1.2-4.5)	
	Parental responsibility		.84		3.02 (0.64; 1.7-4.9)	
	Child control		.66		2.07 (0.57; 1-3.7)	
	Parental efficacy		.54		1.67 (0.41; 1-2.8)	
Perceived social support (SS-A^e^; total)				.95		37.41 (9.78; 23-67)
**Perceived parenting self-efficacy (PSOC^f^)**						
	Satisfaction		.77		23.86 (5.44; 10-36)	
	Efficacy		.79		22.22 (4.07; 7-30)	
	Interest		.63		15.69 (1.95; 6-18)	
**PEEBS^g^**						
	Subjective norms		.79		3.78 (0.50; 1.4-5)	
	Knowledge		.83		3.45 (0.83; 1-5)	

^a^Cronbach *α*, a measure of internal consistency.

^b^SDQ: Strengths and Difficulties Questionnaire.

^c^Cronbach *α* not computed for the Impact scale.

^d^PLOC: Parental Locus of Control.

^e^SS-A: Social Support Appraisals Scale.

^f^PSOC: Parenting Sense of Competence.

^g^PEEBS: Parent Engagement in Evidence-Based Services.

**Table 3:** Bivariate correlation matrix of study variables (N=166).

	1	2	3	4	5	6	7	8	9	10	11
1. Request for resources^a^	X^b^	—^c^	—	—	—	—	—	—	—	—	—
2. Mouse clicks	0.28^d^	X	—	—	—	—	—	—	—	—	—
3. Time spent on resource page^e^	0.32^d^	0.55^d^	X	—	—	—	—	—	—	—	—
4. PRAM^f^ mean total (baseline)	0.48^d^	0.20^d^	0.24^d^	X	—	—	—	—	—	—	—
5. Lack of parental responsibility^g^	0.06	−0.01	0.03	0.07	X	—	—	—	—	—	—
6. Lack of parent control^g^	0.31^d^	0.15^h^	0.23^h^	0.19^d^	0.23^d^	X	—	—	—	—	—
7. Dissatisfaction^i^	−0.25^d^	−0.10	−0.12	−0.26^d^	−0.14	−0.58^d^	X	—	—	—	—
8. Inefficacy^i^	−0.29^d^	−0.13	−0.27^d^	−0.22^d^	−0.20^h^	−0.52^d^	0.48^d^	X	—	—	—
9. Lack of perceived social support^j^	−0.03	−0.04	0.04	−0.09	−0.02	0.32^d^	−0.31^d^	−0.43^d^	X	—	—
10. Subjective norms^k^	0.18^h^	0.09	0.28^d^	0.22^d^	0.03	0.13	−0.07	−0.05	−0.23^d^	X	—
11. Knowledge^k^	−0.10	−0.07	−0.17^h^	0.13	0.05	−0.15	0.06	0.24^d^	−0.10	−0.01	X
12. SDQ^l^ total difficulties	0.30^c^	0.16^h^	0.14	0.22^d^	0.24^d^	0.56^d^	−0.41^d^	−0.33^d^	0.23^d^	0.03	−0.02

^a^Request for resources: 1=yes and 0=no.

^b^*r*=1.

^c^Not applicable.

^d^*P*<.01.

^e^A transformation was applied with log base-10.

^f^PRAM: Parenting Resources Acceptability Measure.

^g^Scales from the Parental Locus of Control measure; higher scores reflect a lower sense of responsibility for and control of the child’s behavior.

^h^*P*<.05.

^i^Scales from the Parenting Sense of Competence scale; higher scores reflect a lower sense of parental satisfaction and efficacy.

^j^Social Support Appraisals Scale total score; higher scores reflect lower perceived social support.

^k^Scales from the Parent Engagement in Evidence-Based Services questionnaire.

^l^SDQ: Strengths and Difficulties Questionnaire.

### Part 1: PRAM and Baseline Validity Measures

After subscale scores were derived for the PRAM based on the EFA, between 1 and 2 low outliers (ie, SD ≥3) were identified for each factor and the total score on both the pre- and postvideo scores and brought to the lower fence. Skewness and kurtosis for each of these scales before and after were within acceptable ranges (<|1.0|).

#### Results of the Factor Analysis

An EFA was conducted using all 18 items; 3 factors emerged with eigenvalues >1. A 3-factor solution was then forced, and the 18 items were subject to principal component analysis with a promax (oblique) rotation. Loadings from the pattern matrix are displayed in Table 4. Factor 1 (9 items) was deemed “Acceptability of Parenting Resources.” Factor 2 consisted of the first 6 items and was deemed “Interest in Learning Parenting Strategies.” Factor 3 (3 items) was deemed “Acceptability of Parenting Websites.” These 3 factors explained 68.4% of the total variance in the measure (factor 1: 51%; factor 2: 9%; factor 3: 8%). Internal consistency of the PRAM scales ranged from good (Cronbach *α*=.89) to excellent (Cronbach *α*=.94). Table 5 contains item and scale descriptives.

Given the small number of items in factor 3, we also examined the reliability of the PRAM with the 3 items from factor 3 omitted and found it to be excellent (Cronbach *α*=.94).

**Table 4:** Parenting Resources Acceptability Measure (PRAM) rotated pattern matrix factor loadings (N=166)^a^.

PRAM item	Factor 1: “Acceptability of Parenting Resources”	Factor 2: “Interest in Learning Parenting Strategies”	Factor 3: “Acceptability of Parenting Websites”
1	0.115	*0.841* ^b^	−0.064
2 (R^c^)	0.181	*0.736*	−0.243
3	−0.009	*0.790*	0.139
4 (R)	−0.058	*0.839*	0.142
5	0.061	*0.735*	0.127
6 (R)	−0.091	*0.858*	0.168
7a	*0.698*	0.064	0.128
7b	*0.693*	0.088	0.059
7c	−0.074	0.186	*0.843*
7d	*0.878*	0.080	−0.173
8a	*0.544*	−0.131	0.450
8b	*0.715*	−0.078	0.221
8c	0.033	−0.045	*0.949*
8d	*0.819*	−0.226	0.179
9a	*0.583*	0.230	0.037
9b	*0.551*	0.230	−0.061
9c	−0.026	0.065	*0.774*
9d	*0.784*	0.169	−0.282

^a^Values reflect factor loadings from the pattern matrix with a promax (oblique) rotation. A 3-factor solution was chosen based on 3 factors with eigenvalues >1. Refer to Multimedia Appendix 1 for the content of the PRAM items.

^b^Italics reflect items that load onto each respective factor.

^c^R: reverse-scored item.

**Table 5:** Bivariate correlations between Parenting Resources Acceptability Measure (PRAM) scales and scale descriptives (N=166)^a^

	PRAM total	Factor 1	Factor 2	Factor 3
Factor 1	0.938	—^b^	—	—
Factor 2	0.880	0.700	—	—
Factor 3	0.715	0.553	0.565	—
Number of items	18	9	6	3
Cronbach *α*	.94	.91	.92	.89
Mean (SD)	3.68 (0.70)	3.54 (0.80)	3.76 (0.78)	3.97 (0.79)
Minimum	1.56	1.12	1.37	1.52
Maximum	5.00	5.00	5.00	5.00

^a^A total of 18 items. All correlations were significant (*P*<.001).

^b^Not applicable.

#### Validity Measures

Table 6 shows PRAM scales and measures of convergent validity. Broadly, the PRAM total score, factor 1, and factor 2 showed similar correlational patterns, whereas factor 3 diverged somewhat. Child maladjustment assessed via the SDQ total difficulties score was positively associated with PRAM factors 1 and 2, such that higher total difficulties related to greater general acceptability of and interest in learning parenting strategies, but was unassociated with factor 3. With respect to parent-level variables, PRAM factors 1 and 2 were positively associated with parental lack of control over the child’s behavior via the PLOC, such that lower perceived control related to greater acceptability of and interest in learning new strategies, but it was unassociated with factor 3. No PRAM scales were associated with parental perceptions of responsibility for their child’s behavior via the PLOC. Interestingly, parental dissatisfaction with the parenting role and parental sense of inefficacy assessed via the PSOC were both negatively associated with PRAM factors 1 and 2 such that lower efficacy and lower satisfaction both related to lower acceptability of parenting resources and education. Dissatisfaction and inefficacy were not associated with factor 3. Knowledge of child treatments via the PEEBS was positively associated with factor 1 such that greater knowledge related to greater general acceptability of parenting resources. Knowledge did not relate to factor 2 or 3. Subjective Norms via the PEEBS was positively associated with factors 2 and 3 such that greater valuation of child treatments being endorsed by others was related to greater interest in learning new strategies and greater acceptability of parenting websites. Subjective Norms did not relate to greater general acceptability of parenting resources (factor 1). Finally, perceived social support assessed via the Social Support Appraisals Scale was negatively associated with factor 3 such that less social support related to lower acceptability of parenting websites. Social support did not relate to factor 1 or 2.

Behavioral outcomes were also positively correlated with parent-reported resource acceptability assessed via the PRAM such that self-reported acceptability aligned with all 3 behavioral measures of engagement: PRAM total by request for resources (*r*=0.48; *P*<.001), time spent on resource page (*r*=0.24; *P*=.002), and mouse clicks (*r*=0.20; *P*=.01; Table 6). Behavioral outcomes showed the strongest associations with factor 2.

**Table 6:** Convergent validity bivariate correlations (N=166).

				PRAM^a^ total		Factor 1^b^		Factor 2^c^		Factor 3^d^
**Child adjustment (SDQ^e^)**										
	**Total difficulties**		0.224^f^		0.221^f^		0.251^f^		0.039	
		Externalizing	0.138		0.143		0.160^g^		0.001	
**Parent variables**										
	Lack of parent control (PLOC^h^)		0.189^g^		0.171^g^		0.227^f^		0.027	
	Lack of parental responsibility (PLOC)		0.074		0.030		0.115		0.088	
	Dissatisfaction (PSOC^i^)		−0.262^j^		0.264^j^		−0.280^j^		−0.039	
	Inefficacy (PSOC)		−0.216^f^		−0.170^g^		−0.307^j^		−0.024	
	Subjective norms (PEEBS^k^)		0.223^f^		0.115		0.269^j^		0.272^j^	
	Knowledge (PEEBS)		0.134		0.169^g^		0.057		0.095	
	Lack of social support (SS-A^l^)		−0.091		−0.034		−0.049		−0.279^j^	
**Behavioral measures**										
	Request for resources		0.476^j^		0.336^j^		0.572^j^		0.374^j^	
	Mouse click count		0.200^f^		0.152		0.254^j^		0.099	
	Time spent on resource page^m^		0.242^f^		0.149		0.322^j^		0.190^g^	
	Prospective resource use^n^		0.359^j^		0.292^j^		0.404^j^		0.223^g^	

^a^PRAM: Parenting Resources Acceptability Measure.

^b^Factor 1: “Acceptability of Parenting Resources.”

^c^Factor 2: “Interest in Learning Parenting Strategies.”

^d^Factor 3: “Acceptability of Parenting Websites.”

^e^SDQ: Strengths and Difficulties Questionnaire.

^f^*P*<.01.

^g^*P*<.05.

^h^PLOC: Parental Locus of Control.

^i^PSOC: Parenting Sense of Competence.

^j^*P*<.001.

^k^PEEBS: Parent Engagement in Evidence-Based Services.

^l^SS-A: Social Support Appraisals Scale.

^m^A transformation was applied with log base-10.

^n^One-month follow-up retrospective report on the use of any parenting resources (n=128).

### Part 2: Group Differences following Experimental Manipulation

#### Acceptability Measure

Four 2 (time: before and after)×2 (condition: standard video and neuroscience-enhanced video) RM ANOVAs were conducted for the composite (mean) of each factor as well as the total score on the PRAM to examine differences between groups from before to after the video manipulation. In line with predictions, for the total score, a significant time-by-condition interaction emerged such that the change in reported acceptability from before to after differed by group (*F*_1,164_=5.202; *P*=.02; *η*^2^=0.031). Follow-up paired-sample *t* tests revealed that acceptability ratings significantly decreased in the standard video condition from before (mean 3.67, SD 0.62) to after (mean 3.60, SD 0.61; *t*_82_=3.107; *P*=.003) but did not significantly change in the neuroscience-enhanced video condition from before (mean 3.69, SD 0.77) to after (mean 3.70, SD 0.74; *t*_82_=−0.530; *P*=.60). For factor 2, dubbed “Interest in Learning Parenting Strategies,” there was also a significant time-by-condition interaction (*F*_1,164_=5.213; *P*=.02; *η*^2^=0.031), with follow-up paired-sample *t* tests showing similar findings (standard video: mean before 3.78, SD 0.72; mean after 3.66, SD 0.73; *t*_82_=2.914; *P*=.005; neuroscience-enhanced video: mean before 3.74, SD 0.85; mean after 3.78, SD 0.77; *t*_82_=−0.675; *P*=.50). There was no significant time-by-condition interaction for factor 1 (*F*_1,164_=2.432; *P*=.12; *η*^2^=0.015) or factor 3 (*F*_1,164_=0.003; *P*=.96; *η*^2^=0.000).

#### Behavioral Measures

##### Overview

Table 7 and Table 8 present descriptives for the pre-post outcome variables and behavioral outcomes by condition, respectively. Behavioral outcomes (ie, requests for resources, mouse clicks, and time spent on the resource page) were all positively correlated (Table 3).

**Table 7:** Descriptives for pre-post outcomes (N=166).

Measure		Standard video (control condition)		Neuroscience-enhanced video (experimental condition)	
		Before^a^, mean (SD)	After^b^, mean (SD)	Before, mean (SD)	After, mean (SD)
**PRAM^c^**					
	Mean total score^d^	3.67 (0.62)	3.60 (0.61)	3.69 (0.77)	3.71 (0.74)
	Factor 1^e^	3.53 (0.73)	3.46 (0.76)	3.55 (0.86)	3.56 (0.87)
	Factor 2^f^	3.78 (0.72)	3.66 (0.73)	3.74 (0.85)	3.78 (0.77)
	Factor 3^g^	3.91 (0.76)	3.89 (0.61)	4.02 (0.82)	3.99 (0.79)

^a^Before viewing the video manipulation.

^b^After viewing the video manipulation.

^c^PRAM: Parenting Resources Acceptability Measure; mean total=mean acceptability of all items (items 1-18).

^d^PRAM before and after mean total scores were strongly positively correlated (*r*=0.92).

^e^Factor 1 (9 items): “Acceptability of Parenting Resources.”

^f^Factor 2 (6 items): “Interest in Learning Parenting Strategies.”

^g^Factor 3 (3 items): “Acceptability of Parenting Websites.”

**Table 8:** Descriptives for behavioral outcome variables (N=166).^a^

Variable			SV^b^ (n=83)		NEV^c^ (n=83)
**Request for parent resources, n (%)**					
	Yes	56 (67.5)		63 (75.9)	
	No	27 (32.5)		20 (24.1)	
Mouse click count, mean (SD)			4.17 (2.99)		4.82 (4.49)
Time spent on resource page (seconds), mean (SD)			55.39 (113.41)		103.21 (188.52)
Log-transformed time spent on resource page, mean (SD)			1.48 (0.44)		1.59 (0.60)

^a^Log-transformed timing was calculated using a base of 10. The parent resource question was posed immediately after viewing the video manipulation and was phrased as follows: “Would you like to learn more about resources for parents on positive parenting practices?” Mouse clicks were counted while viewing the resource page.

^b^SV: standard video.

^c^NEV: neuroscience-enhanced video.

##### Number of Mouse Clicks

A negative binomial regression was conducted to examine group differences in the overdispersed mouse click count data. The number of mouse clicks did not differ significantly by condition (standard video mean 4.17, SD 2.99; neuroscience-enhanced video mean 4.81, SD 4.49; *β*=.145; Wald *χ*^2^_1_ [N=166]=1.3; *P*=.26; 95% Wald CI −0.105 to 0.395).

##### Time Spent on Resource Pages

Owing to high positive skewness and kurtosis, a log transformation (base 10) was applied to this variable. The skewness and kurtosis of the resulting log-transformed variable were acceptable (ie, both <|1|). An independent-sample *t* test was used to test whether the time spent on the resource page differed between groups. There was no significant difference (*t*_150.06_=−1.396; *P*=.17), indicating that the amount of time spent on the resource page did not differ by condition (standard video mean 1.48, SD 0.44; neuroscience-enhanced video mean 1.59, SD 0.60).

##### Expressed Interest in Information on Resources for Positive Parenting Practices

To test for group differences in the tendency to request additional information immediately following the viewing of the video, a chi-square test was conducted. No significant difference was found (*χ*^2^_1_ [N=166]=1.454; *P*=.23), indicating no group differences in this outcome.

### Part 3: PRAM—1-Month Follow-up

#### Use Descriptives

Regarding the subset of parents assessed at follow-up (128/166, 77.1%), the 1-month retrospective reports of resource use were as follows: 60.9% (78/128) reported accessing parenting information on the web, 25% (32/128) reported engaging with at least one book related to parenting, 14.1% (18/128) accessed local parenting resources, and 5.5% (7/128) enrolled in or completed a parenting course. Of the 83 (83/128, 64.8% of the total) respondents who endorsed having engaged with any of the types of parenting resources, 40 (48%) engaged with only 1 type, 35 (42%) engaged with 2 types, 7 (8%) engaged with 3 types, and 1 (1%) engaged with all 4.

#### Predictive Validity

The PRAM total score and each factor at baseline related positively to past-month use of any type of listed parenting resource measured at follow-up (PRAM total: *r*=0.36 and *P*<.001; factor 1: *r*=0.29 and *P*<.001; factor 2: *r*=0.40 and *P*<.001; factor 3: *r*=0.22 and *P*=.01; Table 6). Exploratory analyses examining the associations between PRAM scales and past-month use by resource *type* are shown in Multimedia Appendix 2.

To better understand the relationship between behavioral proxies and actual behavior, exploratory bivariate correlations were conducted between past-month use and mouse click count (*r*=0.01; *P*=.87), log of time spent on the resource page (*r*=0.07; *P*=.41), and request for resources (*r*=0.38; *P*<.001), indicating that only expressed interest in receiving more information on parenting resources was prospectively related to resource use.

#### Group Differences

Within the follow-up completers, RM ANOVAs with Greenhouse-Geisser corrections tested for group differences in change across all 3 time points. There was no significant time-by-condition interaction for PRAM factor 1 (*F*_1.47,185.35_=0.984; *P*=.35), factor 2 (*F*_1.71,215.46_=0.753; *P*=.45), factor 3 (*F*_1.62,204.63_=2.273; *P*=.12), or total (*F*_1.45,182.91_=1.578; *P*=.21), indicating no group differences at the 1-month follow-up.

## Discussion

### Principal Findings

We created and tested a novel measure to assess the acceptability of resources for parenting education or parent skills training. The measure showed good psychometric properties and related to several theoretically relevant measures. Using this measure, we examined whether, in the context of a brief presentation on parenting education, the inclusion of neuroscience information on child development affected parental intent to enroll in parent skills training. The first hypothesis was partially supported; that is, from before to after the video manipulation, parents in the neuroscience-enhanced video condition scored higher on rated acceptability than parents in the standard video condition on PRAM factor 2 (“Interest in Learning Parenting Strategies”) and on the total PRAM score. By contrast, changes in scores did not significantly differ between conditions on factor 1 or factor 3 (“Acceptability of Parenting Resources” and “Acceptability of Parenting Websites,” respectively). Interestingly, the group differences in rated acceptability from before to after were found to be driven by *decreases* in rated acceptability in the standard video condition (in contrast to no significant change from before to after in the neuroscience-enhanced video condition). However, when similar analyses were conducted with the subsample of 1-month follow-up completers, there were no significant group differences across all 3 time points. This may indicate that the impact of neuroscience explanations on acceptability is only short-lived. With respect to behavioral measures (ie, requests for resources, number of mouse clicks, and time spent on the resource page), there were no significant differences by condition. However, all behavioral outcomes indicated levels of engagement in the expected direction, with the neuroscience-enhanced video condition showing nonsignificant higher levels of behavioral engagement. We view the results of this study as a first step toward examining the impact of neuroscience-related information on engagement in prevention and treatment approaches.

The PRAM self-report measure was created for this study to fill the measurement gap in assessing the acceptability of parenting resources or training materials. Three factors emerged, roughly divided in terms of *media format* (ie, *acceptability of parenting resources*, *interest in learning parenting strategies*, and *acceptability of parenting websites*) rather than by facets of *acceptability* (ie, openness, usefulness, and likelihood). It is possible that the differences among various levels of intent to engage are less important than the *ways* in which parents consider engaging. For instance, browsing a website for parenting tips requires very little effort compared with other ways of accessing parenting resources and materials. In line with this, at the 1-month follow-up, most respondents reported having accessed parenting resources in the previous month, and the most commonly accessed resource type was web-based information. Previous studies have shown that most parents find evidence-based parenting information to be acceptable and tend to prefer self-administered formats [41,66]. In this community sample, acceptability ratings appeared favorable across factors and the total score; mean ratings were between *neither agree nor disagree* and *agree*, skewed toward *agree*, and “Acceptability of Parenting Websites” had the highest rated acceptability.

Of note, when the 3 items assessing acceptability of parenting websites (factor 3) were omitted, the PRAM retained excellent internal consistency. Thus, it appears that this measure could be administered as a 15-item measure without a meaningful loss of reliability. However, factor 3 showed multiple unique relations with other variables and, thus, may capture an important swath of parents who have lower levels of interest in information found on mainstream websites. In summary, it is recommended that this measure be modified to include or exclude factor 3 depending on the individual study or intervention purposes.

The PRAM displayed a number of strong psychometric properties. Indeed, the measure earned a passing score on each metric of a set of established criteria [42] used to evaluate measures of acceptability, including reliability, structural validity, criterion (predictive) validity, norms, responsiveness, and usability. Specifically, the PRAM earned ratings of *excellent* on norms (ie, sample size used to establish norms >100), reliability (ie, all Cronbach *α* values ≥.80), and structural validity (ie, N>100, N>7×the number of items, and an EFA explaining >50% of the variance). It earned a rating of *good* on usability (ie, instrument length; between 10 and 50 items) and ratings of *adequate* on criterion validity (ie, medium correlation between the PRAM and another outcome measured in the future) and *responsiveness* (ie, statistically significant change over time on at least a medium-sized sample; N>50). Importantly, each factor of the PRAM and the total score showed small to moderate–strength positive correlations with behavioral proxies of engagement, including prospective associations with self-reported engagement. Thus, the PRAM has predictive validity as a measure of parental engagement. To further explore the PRAM’s responsiveness and criterion validity, it should be used in prospective studies of established interventions that enhance the acceptability of parent skills training.

In our sample, higher ratings of acceptability were associated with greater parent-reported child maladjustment (internalizing and externalizing problems) in addition to greater difficulty in controlling their child’s behavior. Interestingly, higher reports of parenting dissatisfaction and greater feelings of parenting inefficacy were related to *lower* acceptability. Although these findings appear to conflict somewhat with those on child behavioral problems, it may be the case that the parental satisfaction and efficacy constructs may better reflect parental stress or psychopathology than actual child behaviors. Future research should test this hypothesis. Furthermore, parental knowledge of effective child treatments, along with the perceived importance of others’ opinions on child-focused treatments (ie, subjective norms), was associated with greater acceptability. Taken together, our results provide evidence that parents of children who display more problems may be more open to help seeking related to parenting education or skill training. Furthermore, our findings outline parent characteristics that may relate to acceptability, and further work is needed to assess whether these traits, including parental sense of efficacy, satisfaction, knowledge of treatments, and subjective norms, are suitable targets for interventions aimed at increasing initial engagement. Finally, perceived social support related only to acceptability of parenting *websites*; parents who are less socially connected may also have greater mistrust of or less interest in web-based parenting resources touted by experts (eg, the Centers for Disease Control and Prevention) and may benefit from modified engagement methods.

Regarding the experimental manipulation, we found partial support for the hypothesis that self-reported acceptability differed by condition. Specifically, parents who received additional neuroscience information in the video manipulation (neuroscience-enhanced video) did not change their rated interest in learning new parenting strategies from before to after, whereas those in the control condition (standard video) decreased slightly from before to after. Furthermore, at the 1-month follow-up, there were no group differences. It was expected that ratings of acceptability would increase in both conditions, with greater increases in the neuroscience-enhanced video condition. It is possible that parents across both conditions found the video manipulations uncompelling given that no specific parenting intervention or resource was discussed in the video presentations—both videos discussed parent training generally. In addition, given our sample’s skew toward higher educational attainment, it could be that the information presented in the standard video condition was too basic to be of interest, whereas the neuroscience-enhanced video content appealed more to this demographic. It is possible that the pattern of results would differ in a more educationally diverse sample. Despite these unexpected results, this study provides preliminary evidence that the SANE [30,31] can be extended to engage parents—at least initially—in seeking evidence-based parenting resources or education. Interestingly, the only factor of the PRAM that differed between groups was the “Interest in Learning Parenting Strategies” factor and not the 2 factors that included items alluding to specific *media formats* (ie, books, web-based courses, websites, and local resources). Thus, the SANE effect may enhance interest in general parenting education by increasing beliefs that parents can benefit from parenting education or resources but not with respect to specific media formats. Although additional research is needed to test models of parent engagement with and without neuroscience information, findings suggest that including information about the child brain and the effects of parent training on child brain development may be effective in early-stage parent engagement.

With respect to behavioral outcomes, there were no significant differences in outcomes by condition. However, for all 3 variables (ie, mouse clicks, time spent on resource page, and requests for information), the neuroscience-enhanced video had nonsignificantly higher levels of engagement. It is possible that the effects of neuroscience information on parental intent to engage are small, such that this sample size was not large enough to detect a significant difference. In addition, it is possible that the effects of neuroscience information are more related to perceptions of acceptability than to behavioral outcomes indexing intent to engage. All 3 behavioral outcomes were theorized to capture parental *intent to engage*. However, of the 3, only requesting additional resources at baseline was positively associated with prospective use of parenting resources at follow-up. Thus, our behavioral measures may actually reflect other processes, such as general interest or arousal during the survey. It is also possible that behavioral outcomes might have differed at other levels of engagement (eg, actual enrollment and quality of engagement with parent resources). Researchers have highlighted the importance of assessing the effects of engagement techniques at multiple levels of engagement [20,67]. Future work should test the SANE effect on the rates of actual enrollment in a parent skills training program.

### Limitations, Strengths, and Future Directions

It bears noting that the manipulation used was weak, consisting of 37 seconds of additional video content pertaining to basic neuroscience. Although this study was a “proof-of-concept” investigation, future work may achieve greater external validity and larger effects by increasing the dose of the SANE effect (ie, infusing neuroscience information throughout the engagement process). In addition, future work could assess whether the addition of neuroscience explanations to parenting education content enhances outcomes in either engagement or child behavioral improvements. Given the dearth of previous research on this topic, it is difficult to estimate this study’s power to detect true effects. A larger sample size may be needed for a fully powered study.

Following the video manipulation, it is possible that participants found the resources presented to be uncompelling and, thus, general levels of behavioral engagement might have been too low to detect differences across conditions. In this study, we were unable to discern which type of resource parents were most likely to engage with while on the resource page (ie, books, web-based programs, websites, and local resources). Future research could examine which types of resources parents are most likely to behaviorally engage with among a menu of parent resource or training options using methods such as eye tracking and advanced mouse tracking.

Emerging evidence has yielded some support for other theory-driven methods of engagement [28,29], including strategies based on the Health Belief Model [68], which emphasizes attitudes and beliefs about health-promoting behaviors, and the theory of planned behavior and reasoned action [64], which links beliefs and attitudes to perceived social pressure and behavioral capacity to perform an action. The manipulation used in this study—based on the SANE—shared an overlap with the Health Belief Model. Future studies could directly compare engagement strategies based on different theoretical models. In addition, the survey collection methodology may have excluded particularly vulnerable groups of caregivers (eg, single parents caring for multiple young children, those unable to complete a web-based survey at home, or those without access to a computer). Finally, this was a predominantly White, high–socioeconomic status, community sample; SANE effects may differ by race or ethnicity or by socioeconomic status. In addition, SANE effects may differ based on the severity of parent problems, child problems, or parent-child interaction problems. However, with this sample, we were able to examine intent to engage in non–treatment-seeking parents. Most initial parent engagement research focuses on treatment-seeking populations, and influences are examined post hoc instead of a priori. This design allowed for the inclusion of parents across a broad range of behavioral intents to engage with parenting resources.

With respect to future directions, it will be important to test the SANE effect, in addition to the psychometric properties and measure invariance of the PRAM, in more nonparents and non–primary caregivers; caregivers who are treatment-seeking; populations of children with mental or behavioral health diagnoses (ie, clinical samples); and various racial or ethnic, cultural, linguistic, and socioeconomic groups. The effect of presenting neuroscience information related to child development may vary across cultures and across parents with different levels of engagement with mental health care. Indeed, the degree to which these findings may be generalized to other populations—for example, to non-English speakers—is unclear. This study’s lack of linguistic and other cultural diversity is a limitation. Future research should also elaborate and expand on the PRAM. Importantly, the PRAM could be used in research on additional stages of engagement beyond intent to engage (eg, actual enrollment, attrition, and implementation of parenting strategies learned). Future work should also examine the associations between PRAM factors and other constructs, such as parent stress or family empowerment [69]. In addition, the PRAM could be modified for use with different groups (eg, treatment-seeking groups, specific diagnostic groups, and parents with records of child maltreatment) or different prevention contexts (eg, web-based parent interventions and group parenting programs). Finally, this study broadly assessed engagement with parenting education resources; future work should examine the specific effects of engagement interventions on the acceptability of certain *types* or *formats* of resources provided. For example, it is unknown whether there are different factors that are associated with parental engagement in evidence*-*based resources versus other types of parenting resources (eg, a parenting community on social media) or in web-based training programs versus self-help books.

### Conclusions

This pilot study represents a novel merging of 2 literatures: the SANE and parent engagement in education or training. Extant research on the SANE effect was extended by testing this effect on parents. Moreover, a novel method of parent engagement was tested, with preliminary evidence suggesting that the presentation of parenting education and resources may be more compelling in the short term with the inclusion of simple child brain neuroscience information. The findings have implications for public behavioral health efforts that target parents and may advance the state of parenting prevention science. Researchers should continue to strive toward a better understanding of the factors that drive parental engagement, developing and testing novel methods to enhance engagement and engaging caregivers as active stakeholders in this process. This study is one of a handful of studies to experimentally examine initial engagement; it is possible that a combination of variegated strategies, or simple behavioral “nudges” (eg, inclusion of neuroscience information, style and wording of advertisements, and other yet unidentified enhancements), will ultimately prove instrumental in increasing parent engagement in parent education and training. To the best of our knowledge, this study also represents the first psychometrically validated measure to assess initial parent engagement.

## Appendix

Multimedia Appendix 1 Parenting Resources Acceptability Measure.

Multimedia Appendix 2 Intervention videos, resource page, and prospective engagement by resource type.

## Abbreviations

EFA: exploratory factor analysis
PEEBS: Parent Engagement in Evidence-Based Services
PLOC: Parental Locus of Control
PRAM: Parenting Resources Acceptability Measure
PSOC: Parenting Sense of Competence
RM: repeated measure
SANE: Seductive Allure of Neuroscience Explanations
SDQ: Strengths and Difficulties Questionnaire
