# Supplementary material for: Measuring and Enhancing Initial Parent Engagement in Parenting Education: Experiment and Psychometric Analysis
Source: JMIR Pediatr Parent. 2022 Sep 30;5(3):e37449. doi: 10.2196/37449 (PMC9568823; doi:10.2196/37449)
Supplement: Multimedia Appendix 1 [file pediatrics_v5i3e37449_app1.docx]

**Multimedia Appendix 1**

This is a Multimedia Appendix to a full manuscript published in the J Med Internet Res. For full copyright and citation information see <http://dx.doi.org/10.2196/37449>

**Parenting Resources Acceptability Measure (PRAM)**

*Instructions:* Please rate the extent to which you agree/disagree with the following statements.

| 1. I am interested in pursuing a program to learn to use more effective parenting strategies. |
| --- |
| 2. I feel that I do not need any support to increase my parenting skills. (R) |
| 3. I would benefit from learning more effective parenting skills. |
| 4. I have no interest in resources to help me improve my parenting strategies. (R) |
| 5. I would be open to learning new parenting strategies from a child development expert. |
| 6. I do not want to learn any new parenting strategies. (R) |
| 7. I would be open to using the following resources to learn new parenting skills and strategies: |
| 1. A free online course for parents of young children |
| 1. Books written for parents of young children |
| 1. Website with tips for positive parenting techniques |
| 1. Local resources such as agencies and groups for parents of young children |
| 8. It would be useful to use the following resources to learn new parenting skills and strategies: |
| 1. A free online course for parents of young children |
| 1. Books written for parents of young children |
| 1. Website with tips for positive parenting techniques |
| 1. Local resources such as agencies and groups for parents of young children |
| 9. I am likely to use the following resources to learn new parenting skills and strategies: |
| 1. A free online course for parents of young children |
| 1. Books written for parents of young children |
| 1. Website with tips for positive parenting techniques |
| 1. Local resources such as agencies and groups for parents of young children |
| 10. Are there other parenting resources that you would be open to, find useful, or be likely to use? If so, please name them. |

*Note.* Scale: 1 = *Strongly Disagree*, 2 = *Disagree*, 3 = *Neither Agree nor Disagree*, 4 = *Agree*, 5 = *Strongly Agree*. (R) = reverse-scored item.
