# Supplementary material for: Measuring and Enhancing Initial Parent Engagement in Parenting Education: Experiment and Psychometric Analysis
Source: JMIR Pediatr Parent. 2022 Sep 30;5(3):e37449. doi: 10.2196/37449 (PMC9568823; doi:10.2196/37449)
Supplement: Multimedia Appendix 2 [file pediatrics_v5i3e37449_app2.doc]

**Multimedia Appendix 2**

This is a Multimedia Appendix to a full manuscript published in the J Med Internet Res. For full copyright and citation information see <http://dx.doi.org/10.2196/37449>

**Manipulation Videos, Resources Page, & Prospective Engagement by Resource Type**

**Links to Video Manipulation**

***Standard Video (SV):*** <https://youtu.be/AwJ-KVxtDBo>^1^

***Neuroscience-Enhanced Video (NEV):*** <https://youtu.be/dIiDRp3WJqY>^2^

**Supplemental Figure 1**

*Image capture from Neuroscience-Enhanced Video presentation.*

**
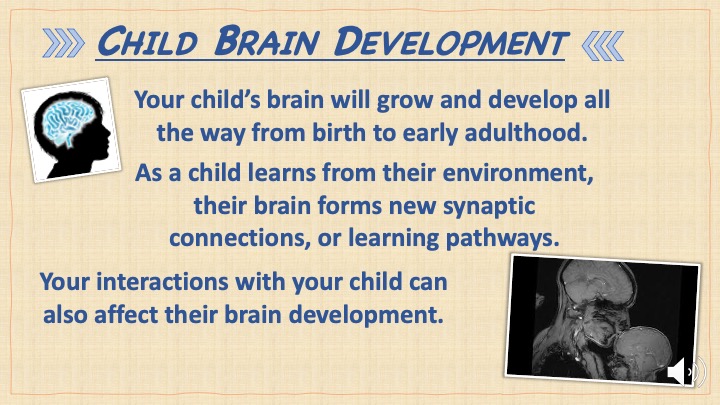
**

**Information Presented on Resources Page Immediately Following Video Manipulation**

***Online Program***

A webpage of a free, 4-week, online, evidence-based, Yale-affiliated parenting course created/taught by Alan E. Kazdin was provided: *Everyday Parenting: The ABCs of Child Rearing* ^3^. Enrollees have the option to purchase the course and earn an official certificate of completion for $49, but users can also access all course content free of charge. An additional parenting course webpage was provided, with courses starting at $79.99 ^4^.

***Self-Help Books***

Evidence-based parenting books included the following: (1) *Parenting from the Inside Out: How a Deeper Self-Understanding Can Help You Raise Children Who Thrive* ^5^, & (2) *The Ten Basic Principles of Good Parenting* ^6^.

***Online Informational Resources***

Web pages from the following evidence-based websites with parenting tips and resources were provided: (1) *Positive Parenting Tips* | Centers for Disease Control and Prevention ^7^, and (2) a list of topics of common parental concerns, and strategies for addressing them from the Child Mind Institute ^8^.

***Local Resources***

A webpage of parent resources on the website of the Early Learning Coalition of the Big Bend Region was provided ^9^. Finally, a community resource directory webpage was provided from the website of Whole Child Leon, a local interdisciplinary organization dedicated to promoting holistic pediatric health across many domains (i.e., socioemotional, economic, spiritual, physical/mental, educational, and environmental) ^10^.

**References**

1. Parent Video (Standard Video [SV])- YouTube. https://www.youtube.com/watch?v=AwJ-KVxtDBo. Accessed February 27, 2022.

2. Parent Video (Neuroscience-Enhanced Video [NEV])- YouTube. https://www.youtube.com/watch?v=dIiDRp3WJqY. Accessed February 27, 2022.

3. Kazdin AE. Everyday Parenting: The ABCs of Child Rearing | Coursera. https://www.coursera.org/learn/everyday-parenting. Accessed November 9, 2019.

4. Online Parenting Skills Classes - Online Parenting Programs. https://www.onlineparentingprograms.com/online-classes/parenting-skills-class.html. Accessed October 21, 2020.

5. Siegel DJ, Hartzell M. *Parenting from the inside out: How a Deeper Self-Understanding Can Help You Raise Children Who Thrive*. 10th Anniv. New York, NY: Jeremy P. Tarcher/Penguin Group; 2013.

6. Steinberg LD. *The Ten Basic Principles of Good Parenting*. New York, NY: Simon & Schuster; 2004.

7. Positive Parenting Tips | CDC. https://www.cdc.gov/ncbddd/childdevelopment/positiveparenting/index.html. Accessed November 9, 2019.

8. Resources for Families | Child Mind Institute. https://childmind.org/audience/for-families/. Accessed November 9, 2019.

9. Parents / A Message From the ELC - Early Learning Coalition. https://www.elcbigbend.org/Parents. Accessed November 9, 2019.

10. Community Resource Directory – Whole Child Leon. https://wholechildleon.org/community-resource-directory. Accessed October 21, 2020.

**Supplemental Table 1**

*Correlations between PRAM and Past-Month Utilization by Resource Type*

|  | PRAM Total | Factor 1 | Factor 2 | Factor 3 |
| --- | --- | --- | --- | --- |
| Books^1^ | .296** | .302** | .246** | .159 |
| Online Information^2^ | .344** | .277** | .396** | .206* |
| Parenting Course^3^ | .200* | .170 | .220* | .106 |
| Local Resources^4^ | .172 | .176* | .120 | .136 |
| Utilization Sum^5^ | .420** | .383** | .410** | .252** |

*Note*. *n* = 128. PRAM = Parenting Resources Acceptability Measure, assessed at baseline; ^1^Item was worded as follows: “In the past month, did you browse, check out, purchase, or otherwise read any parenting-related books? (this can include books we recommended or any other parenting books)”; ^2^ “…did you access or read any information online about parenting? (this can include websites we recommended or any other websites)”; ^3^ “…did you enroll in or complete any online or in-person parenting course? (this can include the courses we recommended or any other parenting course or program)”; ^4^ “…did you access any local parenting resources? (this can include the local resources we suggested or any others)”; ^5^Utilization sum was computed as the total number of types of resources (up to four) engaged with in the previous month.

**P* < .05, ***P* < .01.
